# A Comprehensive Review on Document Image Binarization

**DOI:** 10.3390/jimaging11050133

**Published:** 2025-04-26

**Authors:** Bilal Bataineh, Mohamed Tounsi, Nuha Zamzami, Jehan Janbi, Waleed Abdel Karim Abu-ain, Tarik AbuAin, Shaima Elnazer

**Affiliations:** 1 Software Engineering Department, Faculty of Science and Information Technology, Irbid National University, Irbid 21110, Jordan; 2 Software Engineering Department, College of Computing, Umm Al-Qura University, Mecca 21955, Saudi Arabia; mmtounsi@uqu.edu.sa; 3 Department of Computer Science and Artificial Intelligence, College of Computer Science and Engineering, University of Jeddah, Jeddah 21959, Saudi Arabia; nezamzami@uj.edu.sa; 4 Department of Computer Science, College of Computer and Information Technology, Taif University, Taif 21944, Saudi Arabia; j.gonbi@tu.edu.sa; 5 Applied College, Taibah University, Madinah 41477, Saudi Arabia; wabuain@taibahu.edu.sa; 6 College of Computing and Informatics, Saudi Electronic University, Riyadh 11673, Saudi Arabia; t.aboain@seu.edu.sa; 7 Communication and Electronic Department, Nile Academy for Science and Technology, El Mansoura 35516, Egypt; shaima_elnazer@yahoo.com

**Keywords:** binarization, document analysis, image processing, machine learning, text analysis, thresholding

## Abstract

In today’s digital age, the conversion of hardcopy documents into digital formats is widespread. This process involves electronically scanning and storing large volumes of documents. These documents come from various sources, including records and reports, camera-captured text and screen snapshots, official documents, newspapers, medical reports, music scores, and more. In the domain of document analysis techniques, an essential step is document image binarization. Its goal is to eliminate unnecessary data from images and preserve only the text. Despite the existence of multiple techniques for binarization, the presence of degradation in document images can hinder their efficacy. The objective of this work is to provide an extensive review and analysis of the document binarization field, emphasizing its importance and addressing the challenges encountered during the image binarization process. Additionally, it provides insights into techniques and methods employed for image binarization. The current paper also introduces benchmark datasets for evaluating binarization accuracy, model training, evaluation metrics, and the effectiveness of recent methods.

## 1. Introduction

The process of binarization plays a crucial role in the field of computer vision, particularly in document image analysis [1,2,3]. The primary objective of document image binarization is to improve the legibility and visibility of vital information contained within the document image. This is achieved by classifying pixels in the document image as either black or white, effectively distinguishing the text from the background [2,4]. This binary format is widely preferred for document image recognition and analysis [5,6,7]. Binarization of document images serves various purposes such as noise reduction, text extraction, the removal of unwanted data, reducing the image size in the memory, and preserving the desired information [3,8,9].

The field of document image binarization has garnered significant attention from researchers, as evidenced by the vast number of proposed methods and techniques found in literature reviews [10,11]. The high accuracy of binarized document images is crucial because it makes the images more manageable for tasks like OCR (optical character recognition) and document analysis applications [12,13]. Additionally, there are various software tools available to perform document image binarization, such as OpenCV, Tesseract, and ImageMagick [14,15]. These tools offer several methods and settings to optimize the binarization process for different document image qualities and challenges.

Ordinary binarization methods are typically sufficient for processing high-quality document images. However, certain conditions may degrade the quality of documents during digitization, storage, or physical wear and tear. Degraded document images can be challenging to analyze due to several factors, and older documents may deteriorate over time, causing further degradation. These challenges make the task of document image binarization difficult and often result in poor performance on such images [9,12]. Manual enhancement of these images is often impractical [16,17], emphasizing the importance of developing binarization methods capable of handling the degraded quality and legibility of these images [18].

The objective of this work is to present a thorough and comprehensive survey of document image binarization techniques. It covers the importance of this topic, the issues and challenges that have negatively impacted binarization performance, recently proposed methods and their approaches, the adopted techniques for proposing these methods, benchmark datasets used for evaluation and model training, evaluation protocols commonly employed for assessing binarization performance, and the most recently proposed methods and other techniques used, along with their performance.

This work comprehensively reviews document image binarization, offering a significant contribution through meticulous compilation, systematic analysis, and evaluation of advancements and challenges, making it a valuable resource for both novice researchers seeking a thorough understanding of the field and experienced researchers looking for in-depth insights. In contrast to previous reviews, which often take a narrow approach by primarily presenting and comparing different methodologies, this study provides a broader, integrative framework encompassing traditional algorithms, deep learning, binarization challenges, benchmark datasets, evaluation metrics, performance overviews, and future directions, marking a significant step in document image processing research. The key contributions include the following:

- Comprehensive Overview: Emphasizes the critical role of document binarization in digital document analysis.
- Challenge Identification: Highlights key issues, including camera-captured documents, complex layouts, and historical preservation.
- Methodological Evolution: Traces the progression from traditional thresholding to modern deep learning techniques, evaluating current approaches in terms of effectiveness, robustness, and applicability.
- Benchmark Datasets: Introduces and evaluates datasets for performance assessment and machine learning training.
- Evaluation Metrics: Reviews metrics used in prior studies, advocating a standardized and rigorous evaluation of binarization methods.
- Future Directions: Explores emerging applications and research opportunities, particularly those leveraging deep learning

## 2. Document Binarization Importance

Numerous recent studies have emphasized the importance of image binarization, as it simplifies image data [19]. The binarization process enables quick and easy analysis, processing, and interpretation of image data for various essential applications. For instance, object recognition algorithms often require binarized images as input as it simplifies object detection and segmentation [20]. Additionally, binarization can significantly reduce the image file size, making it more efficient to transmit and store [21]. Binary images are also easier to visualize and analyze than grayscale or color images, particularly for edge detection and morphological operations [22,23].

With the widespread use of smart devices, document binarization is still essential for many new applications. It plays a critical role in document digitization, where paper documents are converted into digital formats using smartphone cameras, reducing the time and resources required for manual data entry [4]. Binarization serves as a preprocessing step for machine learning techniques used in image classification and detection tasks, which often require binary images as input [24]. Moreover, binarization can enhance the clarity of an image, making it easier to read and process. It can extract important features such as edges and contours for further analysis [1,25,26]. Binary images with black text on a white background are the optimal representation of document images and are relied on by OCR systems to accurately analyze, recognize, and extract text from an image [3,8,9].

Binarization is essential for security applications such as document authentication [27] and forgery detection by enabling easier detection and analysis of security features like watermarks and microprinting [27]. It is also used in digital forensics to identify tampering or alterations made to scanned documents [28,29,30]. Binarization enhances the accuracy and reliability of document analysis and textual pattern recognition. The use of deep learning techniques has opened new possibilities in DIAR that rely on accurate binarization as a preprocessing step [31,32,33,34].

Overall, document binarization remains crucial for many new applications today. It is an important process in various fields and applications in image processing. As technology continues to evolve, new applications for document binarization will likely continue to emerge, improving its accuracy and performance, making it an increasingly important tool for document image processing and analysis.

## 3. Overview of Binarization Challenges

Document image binarization presents a significant challenge due to the diverse and often suboptimal conditions encountered during document creation, storage, and digitization. These challenges stem primarily from substantial variations in document content and image quality, as well as the influence of physical and environmental factors. The presence of non-textual elements and handwritten annotations further complicates the process.

This section highlights primary situations that play a major role in creating challenges during the binarization process. These scenarios adversely affect the binarization process and are commonly discussed in the literature. Notable examples include camera-captured documents, documents with complex layouts, and historical manuscripts.

### 3.1. Documents Captured by Camera

Document images that are digitized under perfect conditions are usually binarized simply without any side effects. However, such ideal conditions are not often encountered. Nowadays, smartphones are frequently used for capturing document images. This has led to increased research concerns about document binarization for this type of image [35]. However, camera scanning can be less effective than using a scanner. Camera scanning might not capture the details of the document clearly, resulting in blurry or distorted images. Additionally, the angle and distance from the document can distort perspective, making text alignment and accurate binarization difficult [36,37]. Lighting conditions can also have a negative impact, causing shadows, glare, or reflections on the document, which can affect image quality and make it challenging to determine an appropriate threshold value for binarization. In addition, cameras can capture text surrounded by natural or noisy scenes, which can confuse the binarization process [4,38].

**Figure 1:**
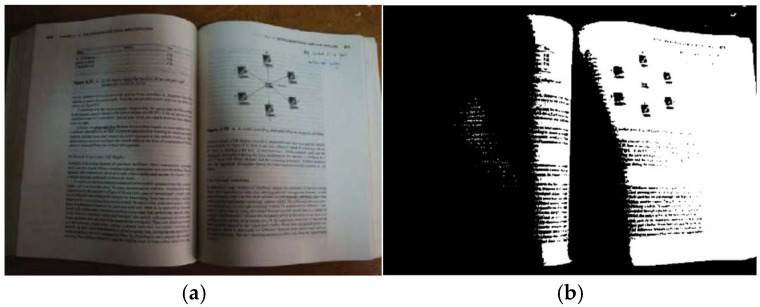
A document image captured by a smartphone camera under challenging conditions (uneven illumination, shadows, skew angle, blurriness, and noisy surroundings). (**a**) The original image and (**b**) the binarization results obtained by using the Otsu method.

Figure 1 shows document images captured under challenging conditions and their binarization results from Otsu [39]. Challenges include uneven lighting and shadows, skewing angles, blurriness, and surrounding noisy scenes.

### 3.2. Documents with Complex Structures

Document images present significant binarization challenges due to their inherent complexity. This complexity arises from various factors, including watermarks, logos, graphics, and diverse text structures such as tables. Furthermore, faint, light, or thin text, which is a common challenge in binarization, makes accurate separation from the background difficult [12]. Modern digital images, such as smartphone screen captures with emojis and stickers, multicolor text, and textured backgrounds, introduce further challenges beyond those faced by traditional grayscale images [40]. Finally, document layout, specifically single- or multi-column formats, adds to the complexity of text detection and, consequently, binarization [41].

It is important to emphasize that although document binarization may yield satisfactory results for documents with complex structures, its capabilities remain inherently limited compared to scene text detectors. Document binarization primarily focuses on separating foreground text from the background by converting the image into a binary representation. Consequently, it cannot locate, classify, or recognize text, as it does not incorporate semantic understanding or contextual information. In contrast, scene text detectors are high-level vision models specifically designed to detect, locate, and recognize text, often within natural environments.

**Figure 2:**
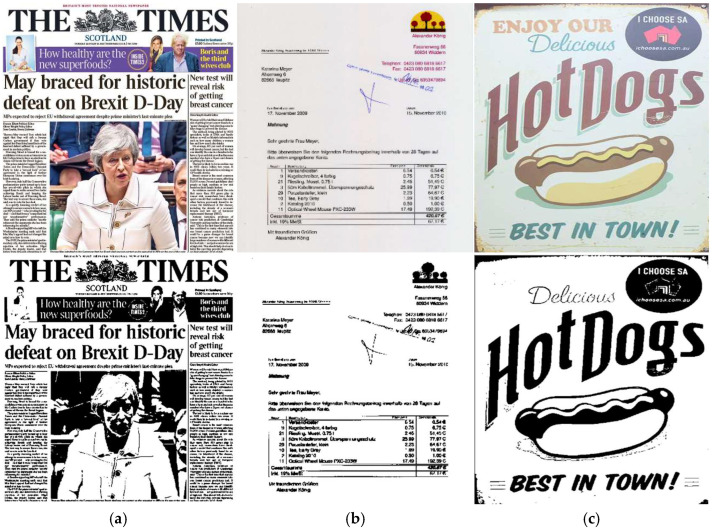
Complex structured documents and their binarization results using the Otsu method: (**a**) multicolor texts and background with a graphical pattern, (**b**) multicolor texts with logos and stamps, and (**c**) multicolored, -sized, and oriented texts with a graphical pattern, all surrounded by natural or noisy scenes.

Figure 2 displays examples of document images with complex structures under challenging conditions, along with their binarization results using the Otsu [39] method. It is clear how these challenges affect the accuracy of binarization.

### 3.3. Degraded and Historical Documents

Document image analysis of ancient and historical documents is essential due to their cultural and historical significance. Binarization plays a crucial role in their preservation, analysis, and dissemination. However, the quality of such documents is highly susceptible to degradation from aging, handling, and storage, leading to physical damage and subsequent binarization difficulties [9,12,13,42,43,44]. Degradation factors such as low image quality, discoloration, fading, and noise further hinder text readability. Additional complexities arise from handwritten and overlapping text, variations in writing styles, and intricate layouts. Furthermore, color variations, folding lines, and fungal spots complicate text extraction. The following explanation illustrates the challenges of converting historical document images to binary images and the impact on accuracy.

- Ink leakage: Ink leakage occurs when ink from one side of the paper bleeds onto the opposite side, leading to overlapping text and uneven background intensities, which complicates binarization [45,46]. This can result in illegible text and dark areas, impeding the application of consistent binarization.
- Fold lines: Folding a document for a long time can leave lines or marks that damage or reduce the quality of crossover texts, posing challenges during the binarization process [44,47,48]. Document folding marks obscure text and creates distortions, leading to missing or illegible text regions, thus complicating binarization.
- Thin text: Degradation of thin text strokes, which is common in historical documents, poses a major challenge to the binarization process and results in weak or lost information [8,49,50]. Binarization algorithms may fail to detect such texts, resulting in incomplete extraction.
- Deteriorated documents: Document deterioration, due to environmental factors such as improper storage and handling, coupled with inherent material instability [51,52,53], can lead to wear and tear, which cause color and contrast variations that hinder binarization algorithms from accurately distinguishing text regions.
- Faded text: Faded text is a common challenge in historical documents. As text fades over time, it becomes lighter, and the density of characters may vary, making it difficult for binarization methods to accurately extract text [54].
- Stains and smudges: Smudges and stains on historical documents significantly impede the process of binarization [55,56]. They can obscure text, cause blurring and distortion, and cause variations in color and contrast. Furthermore, exposure to liquids or moisture can cause ink to bleed, resulting in smudged text.
- Complex layouts and color differences: Historical documents often contain colorful graphics and decorations, which pose significant challenges [57,58,59]. Complex document layouts, denoted by overlapping text, multiple columns, and varying font attributes, greatly complicate the binarization process. Moreover, color differences within a document hinder the algorithm’s ability to distinguish between text and background.
- Contrast variation: Contrast variation caused by factors such as noisy environments and uneven lighting poses significant challenges in the binarization process of historical documents [60,61]. These variations, especially uneven lighting, hinder the accurate designation of text regions.

**Figure 3:**
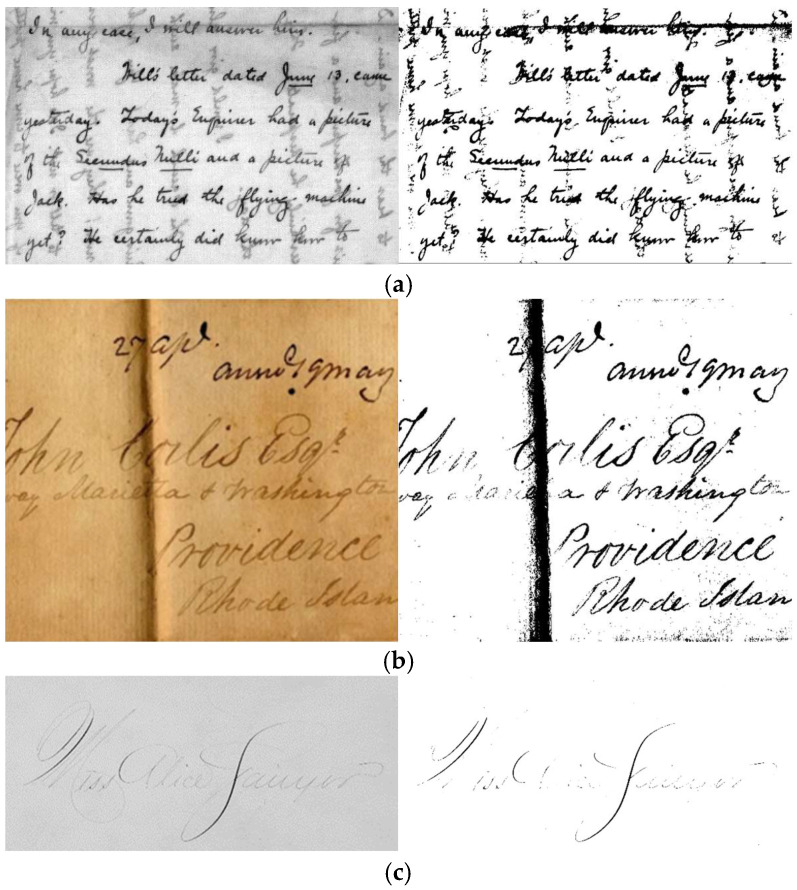

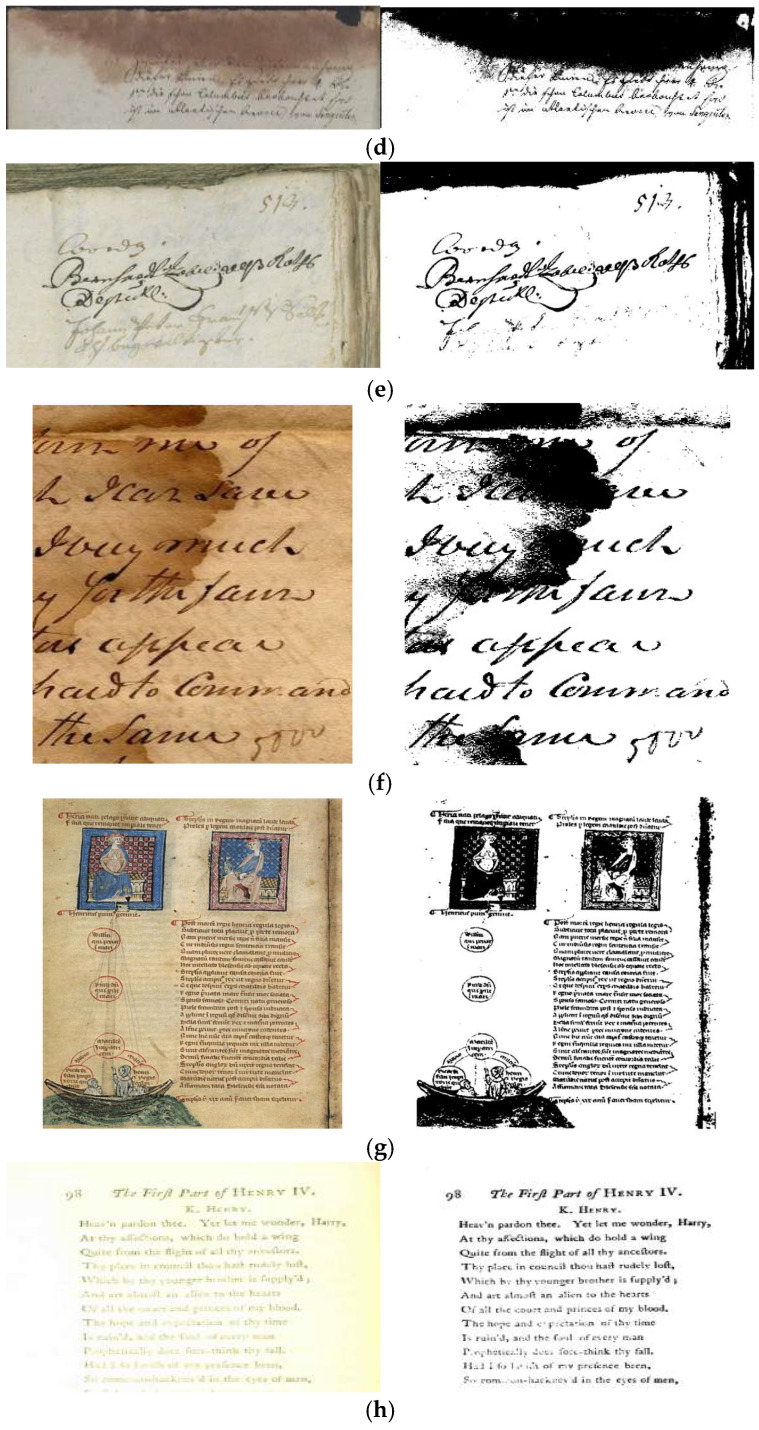
Examples of historical document images with their binarization results obtained by using the Otsu method (**left** the original image and **right** the binarization result): (**a**) ink leakage degradation, (**b**) fold line degradation, (**c**) thin text degradation, (**d**) deteriorated document degradation, (**e**) faded text degradation, (**f**) stain and smudge degradation, (**g**) complex layouts and color variations in an old document, and (**h**) contrast variation degradation.

Figure 3 shows visual examples of historical document image challenges discussed previously, using images from the DIBCO dataset, along with their corresponding binarization results obtained using the Otsu [39] method. Specifically, Figure 3 illustrates (a) an ink bleed challenge, (b) a fold line challenge, (c) a thin text challenge, (d) a document degradation challenge, (e) a faded text challenge, (f) a stain and smudge challenge, (g) complex layouts and color variations in an old document, and (h) a contrast variation challenge.

## 4. Document Binarization Methods

The previous section identified the challenges in the binarization process, and there are different methodologies to address them. Researchers have used a variety of approaches to address the binarization of document images. Previous surveys have reviewed binarization research [9,12,13,15,62,63,64]. Existing methods are broadly classified into thresholding methods, edge-based methods, texture-based methods, clustering-based methods, and machine learning-based methods, as well as hybrid techniques that merge these categories. This section reviews recent methods and their techniques adopted in document binarization.

### 4.1. Threshold-Based Methods

Threshold-based methods are commonly used for document image binarization due to their simplicity and effectiveness compared to other binarization approaches [8,15]. These methods use a threshold value to separate the foreground (text) and background in the image based on whether their intensity values are above or below the threshold value. The threshold can be either global or local [65,66].

- In global thresholding, a single threshold value is applied to the entire image to separate the foreground and background pixels. This approach works well for fine images with uniform backgrounds and foregrounds. However, it may not be suitable for degraded images with variations in foreground and background illumination and intensity values [67,68].
- In local thresholding, the image is divided into sub-images, and multiple threshold values are calculated for each sub-image based on its pixels, allowing the threshold to adapt to changes in pixel values along with the image. This approach is more robust to variations in illumination and intensity values [67,69].

Many benchmark methods for thresholding have been proposed. Otsu’s method [39] is one of the most widely used thresholding methods that was developed by Otsu. It calculates the threshold by minimizing the intra-class variance. The equation is as follows:

σ^2^_w(t) = w1(t) × σ^2^1(t) + w2(t) × σ^2^2(t) (1)

where w1 and w2 are the probabilities of the two classes separated by a threshold value t, and σ^2^1 and σ^2^2 are variances of these two classes. The threshold value that minimizes σ^2^_w(t) is the threshold value for binarization. While simple and computationally efficient, it may struggle with overlapping or poorly defined intensity distribution. Niblack’s method [70] is another benchmark thresholding method that calculates a threshold using the mean and standard deviation of pixel values. The method is defined by the following formula:

T = μ + k × σ(2)

Many researchers have built on Niblack’s principles to enhance document binarization. Works by Bradley [71], Wolf [72], Sauvola [3], Nick, and Bataineh [8] have refined thresholding equations inspired by Niblack’s method. These methods are defined by the following equations:

T_Bradley = μ × (1 − k)(3)

T_Wolf = μ × (1 + k × ((σ/R) − 1))(4)

T_Sauvola = μ × (1 + k × ((σ/R) − 1))(5)

T_Nick = μ + k × sqrt (B + μ^2^) (6)

T_Bataineh = μ − (μ^2^ × σ)/((μG + σ) × (σ + S)) (7)

Here, T is the threshold value, μ is the mean, and σ is the standard deviation of a group of pixel values. The user-defined constant k, along with the size of the local neighborhood, can be adjusted. B is a threshold derived from the local standard deviation, while μG represents the global mean value of all pixel values in the image, S is the scaled standard deviation, and R denotes the dynamic range of pixel values, typically from 0 to 255.

In general, the literature and previous reviews [9,12,15,63] show that global thresholding is a simple and easy-to-implement technique that is generally faster than other thresholding techniques. It works well with images that have uniform backgrounds and consistent lighting conditions. However, it fails with images that have varying backgrounds or uneven illumination conditions. Global thresholding also produces poor results for complex images with multiple objects and varying intensities, leading to over-segmentation or under-segmentation of the objects. In many cases, a single threshold value cannot accurately separate foreground and background regions.

While thresholding remains an essential approach in document image binarization, the limitations of global thresholding have led to reliance on local techniques, as shown in the following. For instance, Mustafa et al. [73] developed a method that utilizes statistical data derived from the local mean and standard deviation to classify each pixel’s neighborhood into background, foreground, and problematic regions characterized by contrast and luminosity issues. Jindal et al. [74] segmented text through background estimation, Otsu thresholding, noise removal, and text enhancement using connected component analysis. Bonny and Uddin [67] introduced a hybrid method that integrates Otsu, Sauvola, Nick, and local adaptive thresholding techniques. Furthermore, Kaur et al. [75] modified the Sauvola binarization technique by dynamically adjusting the window size based on pixel-wise stroke width transformation.

The current literature review highlights local thresholding as the preferred approach due to its advantages. Some recent research focuses on improving existing techniques by integrating preprocessing and postprocessing steps. While local thresholding excels at variable illumination, it can be computationally intensive for large images. Its effectiveness depends on the size of the neighborhood: smaller sizes risk over-segmentation, while larger sizes may cause under-segmentation. Adaptive thresholding also struggles with highly variable illumination and large uniform regions.

### 4.2. Edge-Based Methods

Edge-based binarization tracks foreground text from the background via edge detection [45,76]. This approach is effective for text with well-defined edges but has difficulties in handling complex backgrounds and is sensitive to noise, necessitating preprocessing. Moreover, the computational cost with large images limits its real-time applicability.

Edge detection techniques, such as Canny [77], Laplacian [78], and zero-crossing [76], are commonly used as initial steps in binarization. Despite its popularity, edge-based binarization has seen limited recent development. However, hybrid methods combining edge detection with other techniques have improved performance. For example, [77] uses Canny, k-means, and maximally stable extreme region fusion, while [78] employs background estimation, Laplacian energy analysis, and SWT-based morphological operations. Similarly, methods utilizing time-dependent diffusion and zero-crossing [76] have also improved. However, these methods still face challenges with complex and degraded images and often rely on traditional edge detection and thresholding. Recent studies emphasize incorporating edge detection as a preliminary stage within hybrid deconvolution frameworks to enhance accuracy.

### 4.3. Texture-Based Methods

Texture-based binarization offers advantages in images with complex or heterogeneous backgrounds where edge-based and threshold-based methods have proven insufficient. These methods work by analyzing texture patterns to determine optimal deconvolution thresholds [64]. Previous research has explored different texture analysis techniques for binarization. Run-length histograms were used in early methods [79]. Gabor filters have been applied in the binarization of document images [80]. In addition to Gabor filters, other methods for texture analysis have been investigated, including gray-level co-occurrence matrices (GLCMs) [81], local binary patterns (LBPs) [82], and texture edge descriptors [83].

Recent studies have adopted texture-based approaches for document image binarization. Hsia, Lin, and Chiang [65] used wavelet transforms for frequency decomposition, applying local thresholds and a modified least-mean-square algorithm for background suppression and feature enhancement, respectively. Using inverse transformation and Otsu’s method then yielded a binary image. Sadhad et al. [80] combined Gabor filter texture information with degraded document features for binarizing. Preprocessing included Wiener filtering, and Gabor filters were weighted based on the slant of the text. Postprocessing included morphological operators to reduce artifacts. Susan and Rachna Devi [84] employed sliding window texture matching with a fixed template, generating a distance matrix to which Otsu’s threshold was applied for text area extraction. Lins et al. [85] utilized historical document texture as the primary feature for binarization. Zhang, He, and Guo [86] proposed a nonlinear reaction–diffusion model, using the Perona–Malik equation with a tensor-based diffusion coefficient and nonlinear reaction term for bleed-through document binarization. Ju et al. [87] presents a three-stage GAN approach for binary degraded color document images, focusing on ancient manuscripts. The first stage applies color channel analysis and DWT normalization, the second stage uses channel-specific GANs to extract the foreground, and the third stage integrates local and global predictions. The experimental results show average scores ranging from 75.34 to 79.05.

The literature shows that texture-based methods remain popular for converting document images to binary images due to their ability to preserve image features and integrate with machine learning. However, performance degrades with images with low contrast or low texture. Computing intensity and the need for preprocessing, such as contrast enhancement, are major limitations.

### 4.4. Clustering-Based Methods

Clustering-based document binarization is a technique that uses clustering algorithms to group similar pixels in a document image into foreground and background clusters based on their intensity values [18,88,89,90,91]. Mainly, the k-means clustering algorithm is used widely in binarization in many works such as [77,92,93], and the Fuzzy C-Means algorithm is used in [94,95]. Recent research continues to explore clustering-based document binarization. Bera et al. [18] proposed a hybrid clustering approach, integrating Fuzzy C-means, k-medoids, and k-means for pixel classification. as Also, Kv et al. [96] used the VGG-16 model for image binarization.

The literature shows that clustering algorithms are used in document image binarization due to their compatibility with machine learning trends. They perform well on complex backgrounds and diverse text patterns. However, their performance is limited by poor contrast, uneven illumination, and overlapping text. Sensitivity to algorithm selection and parameter tuning affects performance. Despite these constraints, clustering-based binarization demonstrates notable capabilities in noise reduction and the robust handling of variable document conditions.

### 4.5. Machine Learning-Based Methods

Recent advances in hardware, especially GPUs, have driven machine learning-based document binarization, enabling direct feature learning from annotated datasets and adaptation to diverse document types. Deep learning, particularly convolutional neural networks (CNNs) [97], excels at extracting hierarchical features and adaptive thresholding. In addition, support vector machines (SVMs) [98], neural networks (NNs) [72], and U-Nets [99] show promising results. These methods require training in datasets with paired original form inputs and real binary output images.

Machine learning methodologies have been widely adopted for document image binarization in recent years. Akbari, Al-Maadeed, and Adam [100] employed three prominent convolutional neural networks (CNNs), U-Net, SegNet, and DeepLabv3+, to detect foreground pixels. Dey, Das, and Nasipuri [24] proposed a two-stage framework. The initial stage utilized a generator with variational inference to produce degraded samples, while the subsequent stage employed a CNN-based binarization network trained on these self-generated data. He and Schomaker [6] introduced a T-shaped neural network designed for the dual tasks of binarization and image enhancement. This network incorporated an auxiliary enhancement task to learn image degradation, thereby adapting CNN kernel features for improved binarization.

Yang, Xiong, and Wu [62] presented an end-to-end gated convolution-based network (GDB) for text extraction. This network leveraged gated convolutions to extract stroke features and comprised a coarse sub-network with an edge branch for precise feature mapping, followed by a refinement sub-network for further edge-based refinements. De, Chakraborty, and Sarkar [101] developed a deep learning model for document image binarization using a Dual-Discriminator Generative Adversarial Network (DD-GAN) with Focal Loss. The DD-GAN architecture featured two discriminator networks, and global thresholding was applied to the generated images to produce the final binarized documents. Castellanos, Gallego, and Calvo-Zaragoza [102] combined neural networks with data augmentation to achieve unsupervised document binarization. Suh et al. [103] proposed a two-stage method for color document image enhancement and binarization utilizing Generative Adversarial Networks (GANs). The first stage employed four color-independent adversarial networks to extract foreground information, while the second stage utilized two independent adversarial networks for image binarization, with adversarial loss functions formulated between discriminators and generators. Khamekhem Jemni et al. [104] developed an end-to-end GAN-based architecture to recover degraded documents and enhance readability through a handwritten text recognizer.

Liu et al. [7] proposed a recurrent attention generative model incorporating non-local attention blocks and Spatial Recurrent Neural Networks, validated on two synthetic subtitle datasets. Dang and Lee [105] introduced a multi-task learning approach that learned stroke boundary features and integrated them into the primary binarization task. These learned features were supervised by adversarial loss based on the boundary ground truth to embed expert knowledge into the model. Souibgui et al. [106] proposed a novel encoder–decoder architecture based on vision transformers for enhancing machine-printed and handwritten document images. The encoder processed pixel patches with positional information, and the decoder reconstructed a clean image.

Lihota et al. [107] presented a threshold U-Net model that predicts a low-resolution adaptive threshold map instead of a final binary image. This approach combines classical thresholding techniques with deep learning, achieving similar binary quality to U-Net while offering up to twice the speed and improved memory efficiency. Zhang et al. [108] proposed a lightweight U-Net-based model extended with MobileViT to capture local and global features.

Yang et al. [62] presented a two-stage network that uses gated convolutions to extract selective features, incorporating edge information, and multi-scale operations. Ju et al. [87] presented a GAN-based method that applies discrete wavelet transform (DWT) normalization, trains GANs to extract background for each color channel, and optimizes the output using local models and global.

Du & He [109] proposed a weakly coupled nonlinear diffusion scheme that alternates between restoration and binary. This approach efficiently decomposes images into background and foreground components and demonstrated superior performance compared to eight existing models when tested on degraded document images. Du & He [109] developed a U-Net-based architecture that includes residual, multi-resolution, visual attention, and dilated convolution blocks. This model achieved high accuracy while maintaining a lightweight structure, making it suitable for real-time and mobile applications. Kang, Iwana, and Uchida [110] proposed a U-Net model that utilized pre-trained modular modules and a cascading scheme to address training image scarcity and improve performance. Basu et al. [111] employed U-Net and Pix2Pix for binarizing degraded document images without preprocessing or postprocessing.

The current review indicates a strong shift toward machine learning-based approaches in document image binarization. This trend is attributed to their ability to automate and enhance the efficiency of the binarization process compared to traditional techniques. Furthermore, machine learning models can be designed to handle diverse challenges and binarization requirements by learning from diverse training datasets, thus achieving high levels of accuracy. However, the application of machine learning-based binarization is not without limitations. The primary drawback is the large requirement for labeling training data, which can be a time-consuming, resource-intensive, and expensive task. Additionally, the computational requirements of these methods can be large and require high-performance hardware for models training. Furthermore, the performance of machine learning-based binarization is highly dependent on the quality and diversity of the training data. Consequently, these models may exhibit limited generalization capabilities when faced with unseen data.

In conclusion, the current review highlights image binarization as an active research area. It identifies forty-two significant recent methods (Table 1): threshold-based (five), edge-based (six), texture-based (seven), clustering-based (three), and machine learning-based (twenty-one) methods. Simple threshold methods now often require preprocessing for complex backgrounds. Edge methods excel at text boundaries but struggle with degradation. Texture methods suit complex layouts but need substantial preprocessing. Clustering handles noise but struggles with overlapping and computation. Machine learning, particularly deep learning, is a recent trend offering high accuracy but demanding significant resources. Despite numerous approaches, each has distinct advantages and limitations depending on the document’s characteristics. Further research is needed for consistently high performance across diverse degradations. This review shows that machine learning has received the most attention and has proven reliable for complex binarization. Given advancements in computer vision, especially in pattern recognition under degradation, wider adoption of machine learning in document binarization research appears warranted.

## 5. Benchmark Datasets

Benchmark datasets are essential for developing and evaluating document image binarization. These datasets provide standardized document sets, accompanied by ground truth binary images, thus facilitating the development and improvement of binarization algorithms for better accuracy and efficiency. Specifically, these datasets enable researchers to conduct standard comparative analyses of binarization algorithms, optimize algorithm parameters, and refine machine learning models for document image analysis. They support systematic testing, leading to improved accuracy and efficiency, while fostering the development of new solutions to binarization challenges. As machine learning advances, these datasets remain critical for training and improving models specifically designed for specific document types.

While many datasets have been used in document image binarization research, not all are specifically designed for this purpose. Datasets such as the Tobacco800, the University of Washington-III dataset [12], and the PRImA Layout Analysis dataset [112] lack base binarization images for each original image. However, specialized datasets with base binarization outputs have been developed to address a wide range of binarization challenges. The following section describes some of the main reference datasets used in document duplication.

### 5.1. DIBCO Datasets

**Figure 4:**
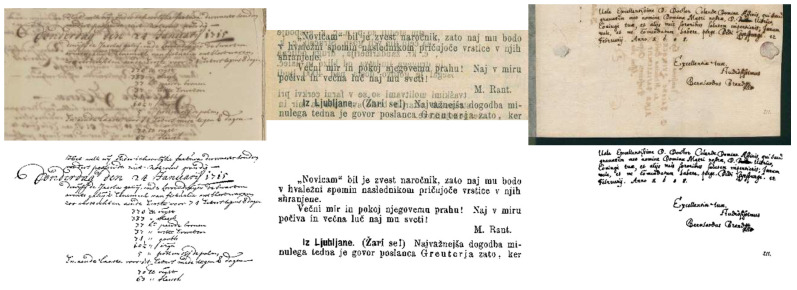
Sample images from the DIBCO dataset along with their corresponding binary ground truth images.

The DIBCO (Document Image Binarization Competition) dataset was first introduced in 2009 [113] and has since become a widely recognized and extensively used benchmark for document image binarization [64,114]. Its primary objective is to provide a standardized set of test images for evaluating the performance of binarization algorithms in a competition format. The H-DIBCO dataset is a specialized subset of DIBCO that focuses on historical documents from various periods. The dataset has been expanded over the years to include additional challenges (listed in Table 2) and covers a broad range of document types [113,115], enabling researchers to test their algorithms’ robustness to real-world challenges. Each image in the dataset includes a binary ground truth. The dataset also includes any expected degradation challenges in historical document images, such as noise, blur, stains and smudges, ink leakage, uneven illumination, and so on (as shown in Figure 4).

### 5.2. Bickley Diary Dataset

**Figure 5:**
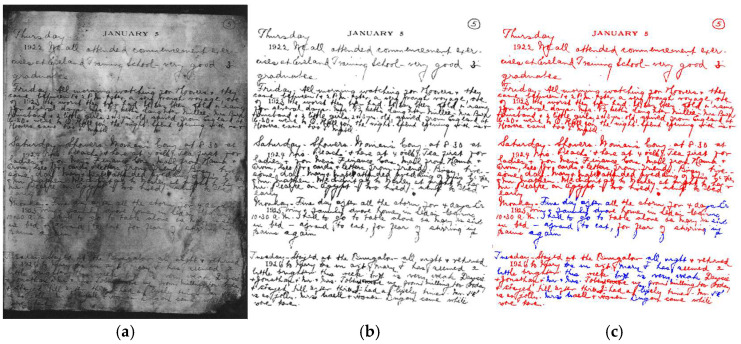
An example of the Bickley Diary dataset: (**a**) the original image, (**b**) the binarized ground truth, and (**c**) a detailed ground truth image.

The Bickley Diary dataset is a benchmark dataset for historical document binarization [116]. The images in the Bickley Diary dataset are taken from a photocopy of a diary that was written about 100 years ago in the 19th century and contain ground truth binary images (as shown in Figure 5). The dataset includes a set of highly degraded document images suffering from different types of degradation, such as water stains and transparent ink. Khitas et al. [116] claimed that the Bickley Diary dataset is more challenging than the DIBCO dataset from a binarization perspective. These benchmark datasets are widely used in the document image analysis community to evaluate and compare the performance of different document binarization algorithms.

### 5.3. LS-HDIB

**Figure 6:**
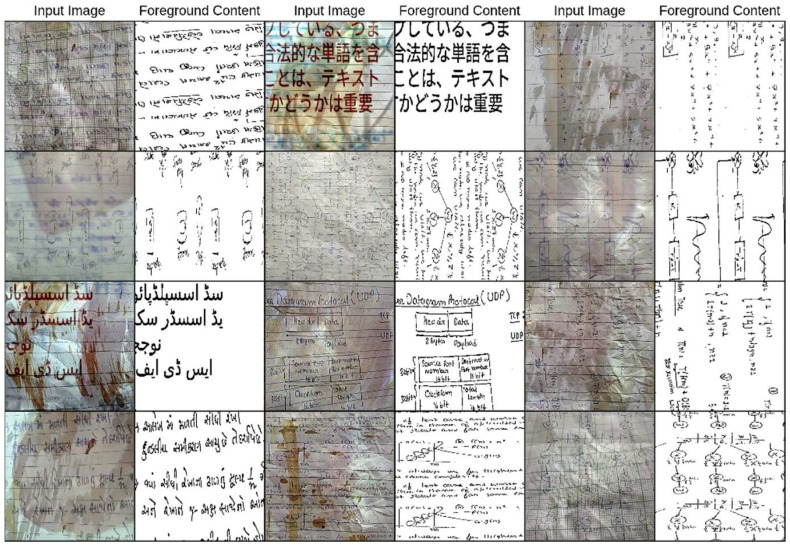
Examples of the LS-HDIB dataset images and their corresponding binary ground truth images.

The LS-HDIB dataset (Large-Scale Handwritten Document Image Binarization) is a recently proposed dataset (2022) aimed at training machine learning methods for document binarization [117]. As the previously mentioned datasets contain a limited number of images that may not be adequate for machine learning training, the LS-HDIB dataset was introduced to address this issue. It is a large-scale dataset containing over one million document images that represent a variety of real-world scenarios. As shown in Figure 6, the dataset provides an accurate ground truth, and the performance of eight different binarization models has been evaluated using this dataset.

### 5.4. PHIBD 2012

**Figure 7:**
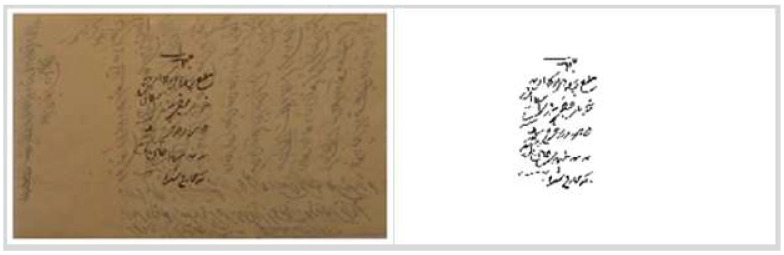
An example image from the PHIBD 2012 dataset and its ground truth image.

The PHIBD 2012 dataset (Persian Heritage Image Binarization) is a dataset of 15 Persian historical document images written in the Arabic language, along with their corresponding ground truths [14,64,118,119]. The images in this dataset suffer from various types of historical document degradation, including bleed-through, faded ink, and blur, among others. Figure 7 shows an example image and its binary ground truth from this dataset.

### 5.5. LRDE DBD

**Figure 8:**
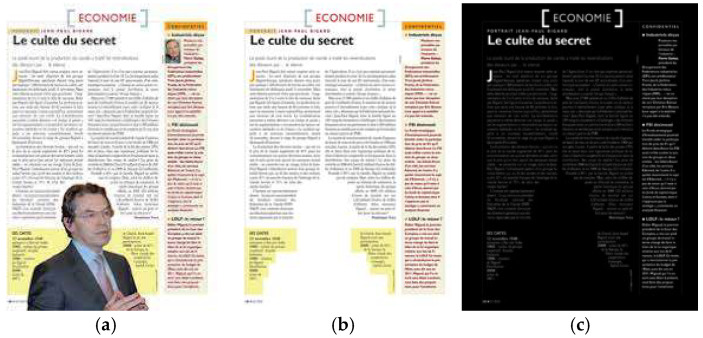
An example from the LRDE DBD, including (**a**) the original document image, (**b**) the clean document image, and (**c**) the ground truth for the scanned document image.

The LRDE DBD (LRDE Document Binarization Dataset) proposed in 2010 consists of 375 printed full-document images with A4 size and 300 dpi resolution, as well as different sizes of fonts and line localizations, taken from the French magazine Le Nouvel Observation [120,121]. It is composed of 125 original documents with a full OCR ground truth, 125 clean documents that contain only text, and 125 scanned documents with slightly degraded text (as shown in Figure 8).

The review of benchmark datasets for binarization reveals that numerous datasets have been proposed specifically for document binarization. Some of these datasets have been recently introduced, indicating that this topic is still relevant and requires further attention in the future. Most of these datasets were designed for evaluation purposes and therefore have a relatively small number of samples, focused on covering a wide range of document degradations. However, the LS-HDIB dataset was proposed for machine learning model training, and it contains around one million document images. These benchmark datasets encompass various document types and languages, enabling research into the development of more robust and accurate document binarization algorithms for a wide range of applications.

## 6. Evaluation and Results

After developing a binarization method, detailed evaluation and comparative analysis are necessary to assess its effectiveness. Previous research on binarization has used various evaluation techniques, including OCR accuracy assessment, segmentation tests, and visual estimation [3,122,123]. However, OCR and segmentation accuracy may not accurately reflect the performance of a binarization method. In addition, visual evaluation alone is inadequate to assess the performance of binarization across diverse user requirements, experimental settings, and conditions [8,45].

To address these limitations, the Document Image Binary Contest (DIBCO) was introduced in 2009 [113,124]. The DIBCO has been instrumental in creating standardized statistical evaluation metrics that compare results to ground truth data [113,115,124,125]. This initiative has provided a standardized basis for evaluating the effectiveness of binarization methods. This section provides an overview of evaluation protocols for document image binarization, examining different evaluation metrics and their importance in determining method performance.

### 6.1. Evaluation Protocols

Effective evaluation of document image binarization methodologies requires a pixel-level comparison between the resulting binary image and its corresponding ground truth. This comparative analysis relies on the calculation of four key metrics:

- True Positives (TPs): The count of pixels correctly identified as the background (black) in the binarized image and matching the ground truth.
- True Negatives (TN): The count of pixels correctly identified as the background (white) in the binarized image and matchings the ground truth.
- False Positives (FPs): The count of pixels incorrectly identified as the foreground (black) in the binarized image, while they are the background (white) in the ground truth.
- False Negatives (FNs): The count of pixels that are incorrectly identified as being in the background (white) in the binarized image, while being in the foreground (black) in the ground truth.

**Figure 9:**
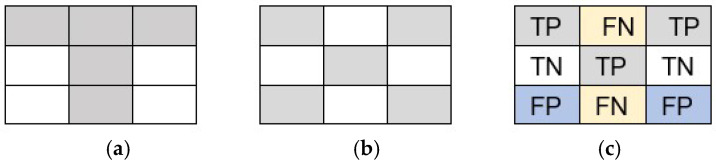
(**a**) The binarized image, (**b**) the corresponding ground truth image, and (**c**) the overlapping pixels between the binarized image and its corresponding ground truth image.

These metrics, derived from a direct pixel-by-pixel comparison of the binarized image with the ground truth binary image, are presented in Figure 9. After calculating the TPs, TNs, FPs, and FNs, the effectiveness of the binarization methods can be evaluated using a set of evaluation metrics. These metrics, including precision, accuracy, recall, F-measure, false F-measure, negative rate measure (NRM), and misclassification penalty measure (MPM), provide quantitative insights into the performance and quality of the binarization results.

- ***Accuracy***: The accuracy metric assesses the overall accuracy of a binary process by computing the percentage of correctly classified pixels in the binarized image when compared to the ground truth [95,126,127]. It offers a comprehensive evaluation of the binarization method’s capability to accurately identify both foreground and background pixels. Accuracy is calculated using the following equation:

Accuracy = (TN + TP)/(TP + TN + FP + FN) × 100% (8)

The accuracy value ranges from 0 to 100, where higher values indicate better performance. Accuracy is simple to compute and widely used, providing a clear overall measure by considering both correctly classified foreground and background pixels. However, it can be misleading in binarized images with dominant background pixels, and it does not capture structural errors.

- ***F-measure***: The F-measure is a metric that offers a comprehensive evaluation of the accuracy and robustness of a binarization method. It combines precision and recall by calculating their harmonic mean, providing a balanced assessment of the method’s effectiveness [105,128,129]. The F-measure is computed using the following formula:

F-measure = 2 × (precision × recall)/(precision + recall)(9)

Here, precision represents the proportion of true positives among the instances classified as positive, and recall (also known as sensitivity) is the proportion of true positives among all positive instances:

Precision = TP/(TP + FP)(10)

Recall = TP/(TP + FN)(11)

The F-measure ranges from 0 to 1, with higher values indicating better performance.

It is a common metric, combining precision and recall into one value. However, it ignores true negatives, is sensitive to ground truth quality, and does not detail error types.

- ***pseudo-F-Measure* (pFM)**: The pseudo-F-Measure (pFM) is a variant of the F-measure used to evaluate the performance of binarization algorithms on document images. Here, the pFM refers to the Fβ measure (weighted F-measure) commonly used in binary classification, which helps balance precision and recall for foreground detection in document binarization [65,103,130]. Unlike the traditional F-measure, the pFM considers the geometric information of the characters in the image. The pFM is calculated as follows:

Pseudo-F-Measure = (1 + β2) × Precision × Recall/(β2 × Precision + Recall)(12)

where β is a parameter that controls the balance between precision and recall. Typically, β is set to 1 to give equal weight to precision and recall. The pseudo-F-Measure ranges from 0 to 1, with higher values indicating better performance. The pFM improves readability evaluation by weighting errors near text, aligning with human perception. However, it is more complex to compute, depends heavily on accurate ground truth, and does not fully account for background preservation or noise removal.

- ***Peak Signal-to-Noise Ratio* (*PSNR*)**: The peak signal-to-noise ratio (PSNR) metric is commonly used in image processing to measure the quality of a reconstructed image by comparing it to the original image [18,131,132]. The PSNR measures the ratio between the maximum possible power of a signal and the power of the distortion that affects the quality of its representation. It is calculated as follows:

PSNR = 20 × log10 (MAX_I) − 10 × log10(MSE)(13)

Here, MAX_I is the maximum value of the image pixels (usually, 255), and MSE is the Mean Squared Error between the original and reconstructed images, which is defined as follows:

MSE = (1/N) × ∑ [i = 1 to N] (I(i) − K(i))2(14)

where i is the original image, K is the reconstructed image, and N is the total number of pixels. The higher values of the PSNR indicate better image quality. The PSNR is a simple and efficient metric widely used in image processing to assess image quality and compare the effects of different binarization methods or compression levels. However, the PSNR does not consider perceptual quality or structural information, which may lead to discrepancies with human perception, particularly when structural text is distorted but pixel differences are minimal.

- ***Geometric-mean pixel accuracy***: The geometric-mean pixel accuracy is a widely used metric in image segmentation to evaluate the accuracy of pixel-level classifications [133]. It provides a comprehensive assessment of segmentation performance by considering both the true positive rate and the true negative rate. The calculation of the geometric-mean pixel accuracy is as follows:

Geometric-mean pixel accuracy = sqrt (sensitivity × specificity)(15)

where sensitivity (true positive rate) measures correctly classified positive pixels, and specificity (true negative rate) measures correctly classified negative pixels. The scale ranges from 0 to 1, with higher values indicating better segmentation accuracy.

Geometric-mean pixel accuracy provides a balanced evaluation of the foreground and background, which is important for imbalanced data, and better reflects document quality for tasks like OCR. However, it can be limited by class imbalance, fine-grained errors, and noise. Its score is less directly linked to the total number of correctly classified pixels, and it treats both classes equally, regardless of their importance. Ground truth quality also impacts its reliability.

- ***Distance Reciprocal Distortion (DRD)***: The Distortion Reciprocal Distance (DRD) measure evaluates the quality of binary image segmentation by measuring the distance between the segmented image and the ground truth, penalizing both false positives and false negatives [65,131]. It is calculated as follows:

DRD = (1/(2 × N)) × (Σd(i) + Σd’(i)) (16)

In this equation, N represents the total number of foreground pixels in the ground truth image. d(i) is the distance from the i-th foreground pixel in the ground truth to its nearest neighbor in the segmented image, while d’(i) is the inverse distance. The DRD ranges from 0 to 1, with lower values indicating higher segmentation accuracy. The DRD assesses binarization by spatial distortion, balancing errors and prioritizing foreground for text. Sensitive to structural issues and context, it is more robust to noise than basic metrics. However, the DRD is computationally intensive, relies heavily on accurate ground truth, and offers limited interpretability.

- ***Negative Rate Metric (NRM)***: The Negative Rate Metric (NRM) is employed to assess binary image results [8,60,134]. It quantifies the rate of correctly classified negative pixels (background) in the output. The NRM is calculated using the following formula:

NRM = TN/(TN + FP) (17)

The NRM value ranges from 0 to 1, with higher values indicating higher accuracy in classifying negative pixels. The NRMSE is a balanced binarization metric considering both false positives and negatives, complementing foreground-focused metrics like precision/recall/F-measure for a broader evaluation. However, relying solely on the NRMSE can be misleading as its numerical value may not always reflect perceived visual quality. For instance, conservative methods might achieve a low NRMSE despite losing significant text.

- ***Misclassification Penalty Metric (MPM)***: The Misclassification Penalty Metric (MPM) is utilized to evaluate binary image results [78,131,134]. It quantifies the penalty incurred when misclassifying foreground pixels as the background, and vice versa. The MPM is computed using the following equation:

MPM = (α × FP + β × FN)/(TP + TN + FP + FN) (18)

Here, α represents the penalty for misclassifying foreground pixels as the background, while β penalizes misclassifying background pixels as the foreground (false positives). The MPM value ranges from 0 to 1, with lower values indicating higher classification accuracy. The MPM reflects text structure by penalizing misclassified pixels based on their distance from ground truth boundaries, making it useful for OCR. However, it is complex, computationally intensive, and depends on accurate segmentation of ground truth. In cases with thin or fragmented characters, the MPM may give low scores despite poor binarization quality.

In conclusion, the effective evaluation of document binarization involves comparing the output to the ground truth at the pixel level, yielding TP, TN, FP, and FN. These form the basis for various metrics such as accuracy, F-measure, pFM, PSNR, GMA, DRD, NRM, and MPM, each with specific advantages and disadvantages. Simpler metrics, including accuracy, precision, recall, F-measure, PSNR, and NRM, offer general assessments, and metrics like NRM can sometimes be misleading when the ground truth is imperfect. Conversely, complex metrics like DRD and MPM offer unique and deeper insights into structure, text quality, and noise, but they are computationally intensive and can sometimes be misleading. Therefore, a balanced evaluation using a combination of these metrics is crucial for a comprehensive understanding of binarization quality, ensuring that both foreground and background aspects are adequately considered.

### 6.2. Evaluation Results

This section presents a comparative analysis of the performance of recent and benchmark binarization methods using the Document Image Binarization Contest (DIBCO) dataset. The evaluation is conducted quantitatively using the F-measure (Fm) and peak signal-to-noise ratio (PSNR) metrics. The F-measure combines precision and recall, providing a balanced evaluation of binarization performance, while the PSNR (peak signal-to-noise ratio) assesses the visual similarity between binarized images and the ground truth, serving as a standard metric within the DIBCO framework. These metrics were selected for their complementary strengths: the F-measure focuses on the accurate detection of foreground text, whereas the PSNR highlights noise suppression and background preservation. Together, they offer a comprehensive evaluation of binarization quality and are widely employed in the relevant literature. Benchmark binarization methods are assessed using standard Python libraries, particularly OpenCV, while recent machine learning-based methods are evaluated based on the results reported in their original publications. The selection of techniques for this quality evaluation was constrained by the unavailability of source code or insufficient details in some original papers, resulting in the deletion of some state-of-the-art methods or reported results for some versions of the DIBCO dataset.

After analyzing sixteen recent methods and four traditional methods presented in Table 3, several of them showed remarkable performance. In general, the machine learning-based methods (ML) showed superior overall performance compared to the other methods. It is difficult to identify the best method due to missing results. However, Quattrini et al. [135] and Ju et al. [87] consistently show higher performance across the available results for multiple DIBCO datasets.

## 7. Conclusions and Discussion

Document image binarization is a crucial preprocessing step for removing unwanted information and retaining textual content. Its importance stems from extensive research conducted to address the challenges affecting its accuracy. This task remains challenging due to the diverse conditions encountered during document creation, storage, and digitization.

After discussing the significance of document image binarization, this paper highlights three primary situations that play a major role in creating challenges during the binarization process. First, camera-captured documents often suffer from inconsistent lighting, blurriness, perspective distortion, and cluttered backgrounds, which make it difficult to apply consistent thresholding methods. Second, documents with complex structures, including watermarks, logos, multicolored or faded text, and complex layouts such as tables or multi-column formats, pose challenges in accurately separating text from the background. Third, deteriorated and historical documents are susceptible to aging effects such as ink bleeds, folding lines, smudges, smears, thin or faded text, and contrast variation. These physical and environmental degradations reduce image clarity and complicate the binarization process. In addition, the presence of handwritten annotations and non-textual elements adds further complexity. Combined, these factors lead to significant variations in image quality, rendering traditional binarization methods ineffective.

Given the wide range and varying degrees of degradation affecting document images, binarization techniques must be adapted accordingly. The choice of a specific binarization method depends on the condition of the document and the nature of the degradation. Consequently, there is currently no single, universally effective binarization method capable of addressing all binarization challenges.

In addition, this review provides a comprehensive analysis of modern binarization methods. These methods are systematically categorized into five types: threshold-based methods, texture-based methods, edge-based methods, clustering-based methods, and machine learning-based methods. The analysis focuses on the fundamental principles of each category, along with their respective strengths and weaknesses. This review highlights the growing dominance of machine learning-based methods over the other categories. While acknowledging their drawbacks, including the need for expensive computational hardware, significant training time, and large, yet still developing, training datasets, machine learning methods show impressive potential for achieving superior results shortly, surpassing other techniques that appear to be fading. However, this does not negate the continued importance of other methods, particularly as potential support mechanisms for machine learning-based techniques.

Furthermore, this review discusses commonly used binarization benchmark datasets. Benchmark datasets play a crucial role by providing standardized and consistent reference data for evaluation, comparison, and algorithm development. Datasets such as DIBCO, Bickley Diary, LS-HDIB, PHIBD 2012, and LRDE DBD represent diverse document types, degradation challenges, and languages, ensuring comprehensive testing in real-world scenarios. While earlier datasets focused on evaluation with limited sample sizes, newer datasets like LS-HDIB address the growing need for machine learning by providing large-scale and diverse training data. These resources not only enhance algorithm performance but also drive innovation in solving complex binarization challenges, such as historical degradation, complex layouts, and handwritten content. As the field evolves, the continued development and expansion of benchmark datasets will remain essential for enhancing the accuracy, efficiency, and adaptability of image binarization techniques in diverse document analysis applications.

This review also discusses evaluation protocols for assessing the effectiveness of document binarization. Effective evaluation requires standardized metrics and comparisons with the ground truth, replacing less reliable traditional methods such as OCR accuracy and visual inspection. The Document Image Binarization Contest (DIBCO) established a framework utilizing key metrics (true/false positives/negatives) to calculate accuracy, precision, recall, and F-measures, alongside image quality metrics like the PSNR, DRD, and MPM. Among these, the F-measure and peak signal-to-noise ratio (PSNR) are widely considered the most reliable due to their balance of simplicity and effectiveness. The F-measure provides a balanced evaluation by combining precision and recall, while the PSNR evaluates the visual similarity between binary images and the ground truth and serves as a standard metric within the DIBCO framework.

Finally, this paper presents a comparative analysis of the performance achieved by 20 binarization methods evaluated on the DIBCO dataset. The analysis includes both recent machine learning-based and traditional algorithms. The results indicate that recent methods utilizing machine learning models generally outperform traditional binarization techniques. In particular, the machine learning methods proposed by Ju [87] and Quattrini [135] demonstrate notably strong performance. The strong performance of Ju [87] and Quattrini [135]’s methods likely results from machine learning’s automatic feature learning, adaptability to various degradations, and contextual understanding. They likely excel even in the face of common complex issues like uneven lighting, noisy backgrounds, fading, and bleed-through, given relevant training data. Limitations include reliance on large, diverse datasets, computational cost, and potential generalization issues with entirely new degradations or document styles.

While several methods show significant performance, they typically address specific degradation issues. No single method effectively handles all types of document degradation, highlighting a compelling need for more research to find a robust generalized method that can handle all forms of binarization challenges. Future research should prioritize developing such a method to address the diverse challenges in document binarization. Based on this review, we propose two main recommendations for future work:

- There is a critical need for larger, more varied, and comprehensive datasets that cover various languages, scenarios, and challenges for training machine learning-based binarization models.
- While earlier methods combined diverse approaches, recent trends favor machine learning. We propose exploring hybrid methods by integrating machine learning with traditional techniques like enhancement, edge detection, and filtering, alongside other binarization types, to leverage their combined strengths for improved accuracy and robustness.

## Figures and Tables

**Table 1:** Recent binarization methods.

Author(s) (Year)	Method Used	Advantages	Disadvantages
*Thresholding-based methods (5 methods)*			
Bonny and Uddin (2020) [67]	Integration of Otsu, Sauvola, and Nick	Combines strengths of different methods	Slow and complex
Kaur et al. (2020) [75]	Dynamic Sauvola with adaptive window size	Better binarization via stroke width adjustment	Complex window size estimation
Bardozzo et al. (2021) [68]	Global techniques for uniform background	Fast and effective for clean documents	Fails with non-uniform illumination
Jindal et al. (2021) [74]	Background estimation + Otsu + CCL	Improved text segmentation	High computational cost
Mustafa et al. (2022) [73]	Classifies regions via local stats	Good under contrast and brightness variations	Requires preprocessing and tuning
*Edge-based methods (3 methods)*			
Guo et al. (2020) [76]	Zero-crossing + time-dependent diffusion	Separates text using local features	Slow and less effective on degraded images
Xiong et al. (2021) [78]	Laplacian energy + SWT + background estimation	Edge detection using structured morphology	Computationally heavy; needs preprocessing
Wu et al. (2022) [77]	Canny edge detection	Captures sharp boundaries well	Fails on blurred, low-contrast, or noisy images
*Texture-based methods (7 methods)*			
Yang and Yan (2000) [79]	Run-length histograms for texture analysis	Works well on varied backgrounds	Struggles with complex documents
Hsia et al. (2020) [65]	Wavelet transforms + local thresholds + LMS	Effective for complex backgrounds	Extensive preprocessing and postprocessing
Susan et al. (2020) [84]	Sliding window texture matching + Otsu	Good text regions in complex layouts	Fixed template may not suit all document layouts
Zhang et al. (2020) [86]	Reaction-diffusion model + Perona-Malik + tensor diffusion	Excellent for bleed-through artifacts	Computationally expensive; not suitable for all document types
Ju et al. (2022) [87]	GAN + foreground extraction + integration	Advanced for degraded, ancient manuscripts	Complex and time-consuming
Lins et al. (2022) [85]	Use texture as a feature	Ideal for historical docs	Limited generalizability
Bernardino et al. (2024) [81]	Gray-level co-occurrence matrices	Effective for complex backgrounds	Decreases performance with low contrast/noise
*Clustering-based methods (2 methods)*			
Bera et al. (2021) [18]	Hybrid of clustering methods	Effective in complex backgrounds	Complex and computationally expensive
Kv et al. (2023) [96]	VGG-16 integrated with clustering	Suitable for modern document images	High computational cost and training data requirements
*Machine-learning methods (20 methods)*			
Ghoshal & Banerjee (2020) [98]	SVMs for features and classification	Effective binary classification	Sensitive to features and tuning
Basu et al. (2020) [111]	U-Net and Pix2Pix	Good for degraded docs	Struggles with noise
Akbari et al. (2020) [100]	U-Net, SegNet, and DeepLabv3	Robust and handles text extraction well	Requires lots of training data
De et al. (2020) [101]	DD-GAN with focal loss	Good for degraded documents	GANs are hard to train
Liu et al. (2020) [7]	Recurrent attention GAN with Spatial RNNs	Handles degradation well	Needs large datasets and has high computational requirements
Zhao et al. (2021) [99]	U-Net	Good for complex docs	High computational cost
He et al. (2021) [6]	T-shaped neural network	Enhances image quality	Requires large datasets
Kang et al.(2021) [110]	U-Net with pre-trained modular cascade	Better generalization; less training data	Module selection critical
Castellanos et al. (2021) [102]	NN with data augmentation	Works on diverse data; unsupervised	Sensitive to data variability
Dang & Lee (2021) [105]	Multi-task learning with stroke boundaries + adversarial loss	Embeds expert knowledge	Risk of overfitting to strokes
Dey et al. (2022) [24]	Two-stage CNN + variational inference	Adaptable to degradation	Complex; may not handle extreme degradation
Suh et al. (2022) [103]	Two-stage GAN	Robust to variations	Needs careful training
Khamekhem (2022) [104]	End-to-end GAN	Good on degraded documents	Resource-heavy training
Souibgui (2022) [106]	Vision encoder–decoder	Good on degraded documents	Large datasets and GPU
Ju et al. (2022) [87]	GAN with wavelet	Good on degraded documents	High computational load
Yang et al. (2023) [62]	Gated convolutions	Precise edge mapping	Slow on large documents
Lihota et al. (2024) [107]	Threshold U-Net	Memory-efficient; fast	Sensitive with resolution
Zhang et al. (2024) [108]	U-Net + MobileViT	Lightweight; works in real time	Sensitive with large documents
Yang et al. (2024) [62]	Gated convolutions	Works in diverse styles	Needs high-quality data
Du & He (2024) [109]	Nonlinear diffusion	Efficient and accurate	Struggles with noise

**Table 2:** The list of the different versions of the DIBCO dataset.

Version	Description
DIBCO 2009 (https://users.iit.demokritos.gr/~bgat/DIBCO2009/, accessed on 1 January 2025)	5 degraded handwritten documents and 5 degraded printed documents
DIBCO 2010 (https://users.iit.demokritos.gr/~bgat/H-DIBCO2010/, accessed on 1 January 2025)	10 handwritten document images
DIBCO 2011 (http://utopia.duth.gr/~ipratika/DIBCO2011/, accessed on 1 January 2025)	8 printed and 8 handwritten images
DIBCO 2012 (http://utopia.duth.gr/~ipratika/HDIBCO2012/resources.html, accessed on 1 January 2025)	8 handwritten images and 8 printed images
DIBCO 2013 (http://utopia.duth.gr/~ipratika/DIBCO2013/benchmark, accessed on 1 January 2025)	8 handwritten images and 8 printed images
DIBCO 2014 (http://users.iit.demokritos.gr/~bgat/HDIBCO2014/benchmark, accessed on 1 January 2025)	10 handwritten images without any printed images
DIBCO 2016 (https://vc.ee.duth.gr/h-dibco2016/, accessed on 1 January 2025)	10 handwritten images with different sizes and resolutions
DIBCO 2017 (https://vc.ee.duth.gr/dibco2017/benchmark/, accessed on 1 January 2025)	10 handwritten images and 10 printed images
DIBCO 2018 (http://vc.ee.duth.gr/h-dibco2018/benchmark/, accessed on 1 January 2025)	10 handwritten documents with representative degradations
DIBCO 2019 (https://vc.ee.duth.gr/dibco2019/, accessed on 1 January 2025)	10 historical printed and 10 historical handwritten document images

**Table 3:** Document binarization results on DIBCO dataset.

			DIBCO									
Method	Category	Metrics	‘09	‘10	‘11	‘12	‘13	‘14	‘16	‘17	‘18	‘19
Niblack [70]	Thresh	FM	-	-	70.4	-	71.4	86.0	72.6	51.2	41.2	51.5
		PSNR	-	-	12.4	-	13.5	16.5	13.3	7.7	6.8	10.5
Otsu [39]	Thresh	FM	78.6	85.4	82.1	75.1	80.0	91.6	86.6	77.7	51.5	47.8
		PSNR	15.3	17.5	15.7	15.0	16.6	18.7	17.8	13.9	9.7	9.1
Sauvola [3]	Thresh	FM	85.4	75.2	82.1	81.6	82.7	84.7	84.6	77.1	67.8	51.7
		PSNR	16.4	15.9	15.7	16.9	17.0	17.8	17.1	14.3	13.8	13.7
Bataineh [8]	Thresh	FM	85.1	80.4	83.5	81.9	82.4	84.2	83.7	78.7	61.3	-
		PSNR	16.2	16.6	16	16.9	17.0	17.0	16.7	14.5	12. 9	-
He [136]	ML	FM	94.9	93.9	95.3	92.9	95.7	97.7	91.1	92.7	92.2	-
		PSNR	20.5	21.2	21.5	22.9	23.0	23.9	19.2	19.2	20.1	-
Kang [110]	ML	FM	96.7	-	95.5	95.2	95.9	97.1	93.1	91.6	89.7	-
		PSNR	20.9	-	19.9	21.4	23.0	22.4	19.2	15.9	19.4	-
Yang [62]	ML	FM	93.6	95.5	94.6	95.4	95.9	97.6	89.9	91.3	90.8	73.9
		PSNR	20.4	22.3	20.7	22.3	22.9	23.7	18.9	18.3	19.7	14.8
Suh [103]	ML	FM	93.3	93.9	93.4	94.5	94.8	96.2	91.1	91.0	91.9	70.6
		PSNR	19.7	21.2	20.0	21.8	21.8	21.8	19.3	18.4	20.0	14.7
Souibgui [106]	ML	FM	-	-	94.2	95.1	-	-	-	92.5	90.6	-
		PSNR	-	-	20.6	22.0	-	-	-	19.1	19.5	-
Bera [18]	Cluster	FM	-	-	-	-	-	-	90.4	83.4	76.8	72.9
		PSNR	-	-	-	-	-	-	18.9	15.5	15.3	14.5
Dang [105]	Edge	FM	-	-	-	-	96.0	-	-	92.1	91.3	-
		PSNR	-	-	22.1	-	23.1	-	-	18.7	19.8	-
Huang [137]	ML	FM	-	-	-	-	-	-	89.7	90.7	91.8	-
		PSNR	-	-	-	-	-	-	18.9	17.9	19.8	-
De [101]	ML	FM	-	-	-	-	92.1	96.9	87.6	91.0	88.3	-
		PSNR	-	-	-	-	20.7	22.7	18.1	18.3	19.1	-
Basu [111]	ML	FM	-	-	-	-	93.6	95.4	89.6	92.3	89.9	60.1
		PSNR	-	-	-	-	21.3	22.4	19.0	18.9	19.4	12.4
Hsia [65]	Texture	FM	82.7	79.7	85.6	81.1	87.7	86.9	85.7	84.2	-	-
		PSNR	15.9	15.4	17.8	17.0	18.1	17.8	18.5	15.2	-	-
Detsikas [138]	ML	FM	95.7	-	94.68	-	-	97.55	90.8	92.2	85.9	-
		PSNR	21.42	-	21	-	-	23.62	19.2	18.7	18.2	-
Ju [87]	ML	FM	-	-	94.22	-	94.6	96.6	91.8	91.3	92.9	-
		PSNR	-	-	20.54	-	22.0	22.23	19.7	18.6	20.4	-
Quattrini [135]	ML	FM	-	96.4	96.03	97	96.7	98.19	91.3	93.8	93.	-
		PSNR	-	23.4	22.26	24.3	24.2	25.18	19.7	19.6	20.9	-
Lihota [107]	ML	FM	-	-	-	-	-	-	-	88.6	-	-
		PSNR	-	-	-	-	-	-	-	17.5	-	-
Zhang [108]	ML	FM	-	-	-	96.4	-	-	-	93.2	90.59	65.92
		PSNR	-	-	-	23.3	-	-	-	19.3	19.52	15.25
